# Species and characteristics of volatile organic compounds emitted from an auto-repair painting workshop

**DOI:** 10.1038/s41598-021-96163-4

**Published:** 2021-08-16

**Authors:** M. Y. Song, H. Chun

**Affiliations:** Department of Climate & Environmental Research, Seoul Institute of Technology, Seoul, 03909 Korea

**Keywords:** Environmental sciences, Chemistry

## Abstract

Volatile organic compounds (VOCs) are secondary pollutant precursors having adverse impacts on the environment and human health. Although VOC emissions, their sources, and impacts have been investigated, the focus has been on large-scale industrial sources or indoor environments; studies on relatively small-scale enterprises (e.g., auto-repair workshops) are lacking. Here, we performed field VOC measurements for an auto-repair painting facility in Korea and analyzed the characteristics of VOCs emitted from the main painting workshop (top coat). The total VOC concentration was 5069–8058 ppb, and 24–35 species were detected. The VOCs were mainly identified as butyl acetate, toluene, ethylbenzene, and xylene compounds. VOC characteristics differed depending on the paint type. Butyl acetate had the highest concentration in both water- and oil-based paints; however, its concentration and proportion were higher in the former (3256 ppb, 65.5%) than in the latter (2449 ppb, 31.1%). Comparing VOC concentration before and after passing through adsorption systems, concentrations of most VOCs were lower at the outlets than the inlets of the adsorption systems, but were found to be high at the outlets in some workshops. These results provide a theoretical basis for developing effective VOC control systems and managing VOC emissions from auto-repair painting workshops.

## Introduction

Volatile organic compounds (VOCs) in the atmosphere produce photochemical ozone (O_3_) and other oxidants, which increase the atmospheric oxidizing ability^1^, and are also responsible for the formation of secondary organic aerosols (SOAs)^2,3^. Both O_3_ and SOAs have the potential to adversely impact human health^4–6^. VOCs have been investigated with respect to both environmental and human health; in studies concerning the latter, certain VOCs have been categorized as toxic air pollutants. Some VOCs have been known to be carcinogenic; chronic exposure to VOCs has been known to cause cancer^7^. Furthermore, some VOCs exhibit harmful effects on the central nervous system, respiratory system, liver, etc., resulting in fatigue, headaches, and breathing difficulty^8^.

VOCs are continuously emitted from large-scale industrial complexes and urban areas that have facilities such as laundry, printing, restaurants, and auto-repair painting, among others^9,10^. Seoul is the capital and largest metropolis of South Korea, with a population of 9.7 million, and several small-scale VOC emission sources are present in the urban areas^11^. The Seoul Metropolitan Region comprises Seoul, Incheon, and Gyeonggi, and the concentrations of O_3_ in the region have been reported to be higher than those in any other region in Korea, indicating a continuous increase^12^. Recently, the Korean government has initiated supporting control systems of small-scale VOC emission sources and recommended the use of water-based paints for workplaces larger than a certain size in order to reduce the amount of VOCs in organic solvent emission facilities. In Seoul, “organic solvents” accounted for 82% of the total VOC amount, and “road transportation pollutants” and “non-road transportation pollutants” accounted for 11 and 3%, respectively, in 2017^11^. Specifically in the category of “organic solvent application,” VOC emissions from “painting facilities” account for approximately 60% of the total organic solvent emission, with “printing industries” and “laundry facilities” at 24 and 14%, respectively^11^. Therefore, the management of VOC emissions from the “painting facilities” in Korea is essential.

Previous studies have focused on VOC emission testing using a chamber^13–15^ or the VOCs emitted from large-scale industrial sources^16–19^, with the impact of the emitted VOCs on the environment alone being considered. In general, an industrial complex and a measurement site of interest are selected, and ambient VOC concentrations are measured through real-time monitoring or sampling to determine the level and temporal variability of pollution. In addition, the VOC data obtained are used for profiling, grouping, and analysis of individual compounds, which are then further studied to determine tracer indicators to identify their concentration patterns under different environmental conditions.

Kamal et al.^20^ conducted a study assessing the potential health risks to workers, but detailed information on VOCs emitted from auto-repair painting facilities, as a small-scale VOC emission source, has not been provided. VOCs are either directly emitted in large quantities from the outlets of painting facilities or via diffusion from the solvent content in paints^21^. Various paints and complex processes (pretreatment, primer, base coat, top coat, drying, and polishing) exist in auto-repair painting, and some of the VOC emissions are likely to be unaccounted for because of the lack of specific measurements of VOC chemical profiles from the painting facilities. This makes identifying the main VOC emission sources and devising efficient VOC emission control strategies difficult. Hence, a control strategy for reducing VOC emissions from the source facilities, including auto-repair painting facilities, is essential for reducing the ambient O_3_ levels as well as SOA formation^22,23^.

To effectively control the indoor and outdoor pollutants emitted from painting facilities, we performed a chemical profile analysis of the VOCs emitted from the main painting workshop in an auto-repair painting facility at Korea. In particular, we compared the VOC species emitted from the use of oil- and water-based paints and analyzed them by grouping into BTEX (benzene, toluene, ethylbenzene, and xylene), ketone, and O_3_ precursor groups. In addition, we measured the concentrations of the individual VOCs at the inlet and outlet of the adsorption systems installed in the auto-repair painting booths. This study will help understand the characteristics of VOCs emitted from small-scale auto-repair painting facilities and support development of efficient VOC emission control strategies.

## Results

### Total VOC (TVOC) concentration: comparison between workshops and interpretation based on the experimental setup

TVOC (sum of TO-14 VOCs) concentrations ranged from 5069 to 8058 ppb in the main workshop and 24–35 VOC species were detected, including precursors of O_3_ formation (Supplementary Table S1). Table 1 shows the 16 VOC species that were detected to be emitted in the highest concentrations from the top coat workshop. The observed concentrations of the individual VOCs ranged from 0 to 3378 ppb (butyl acetate had the highest concentration in workplace E); the differences between the workshops were mainly influenced by conditions such as the amount of paint used in each facility, operator characteristics, and type of paint^24^. For all workshops, butyl acetate, toluene, ethylbenzene, m,p-xylene, o-xylene, and 1,2,3-trimethylbenzene were the most commonly detected species, and workshops A and C had lower concentrations of these compounds than the other workshops. Workshops A, C, and E used water-based paint, whereas workshops B and D used oil-based paint. According to Can et al.^25^, higher VOC concentrations are found in painting workshops using oil-based paints than in those using water-based paints. The species composition of VOC emission is detailed in the following sections.

**Table 1:** Concentrations of the measured volatile organic compound (VOC) species in different workshops of auto-repair painting (unit: ppb).

Component	Workshop A	Workshop B	Workshop C	Workshop D	Workshop E
Butyl acetate	3256	2449	3054	2581	3378
Methyl chloride	0.000	177.6	38.01	30.61	211.9
Acrylonitrile	0.000	0.000	0.000	43.86	0.000
1-Butene	55.66	60.42	14.30	16.10	244.2
Toluene	118.6	192.6	246.5	689.8	613.8
Octane	8.756	4.759	8.465	8.381	14.08
Ethylbenzene	160.4	597.9	215.1	325.4	599.2
m,p-Xylene	389.1	1789	367.2	1371	383.1
o-Xylene	143.6	986.4	132.5	507.7	151.5
n-Propylbenzene	70.85	123.2	61.98	31.13	27.39
m-Ethyltoluene	209.9	316.3	173.0	79.31	66.12
p-Ethyltoluene	69.28	164.0	61.77	40.72	100.4
1,3,5-Trimethylbenzene	94.45	190.4	78.22	56.81	83.04
o-Ethyltoluene	70.92	138.4	58.88	32.58	56.46
1,2,3-Trimethylbenzene	244.9	514.2	194.1	122.5	316.5
1,2,4-Trimethylbenzene	80.77	239.3	66.98	45.59	137.6

### Emission of individual VOCs and their concentrations and proportions: comparison between workshops and interpretation based on the experimental setup

**Figure 1:**
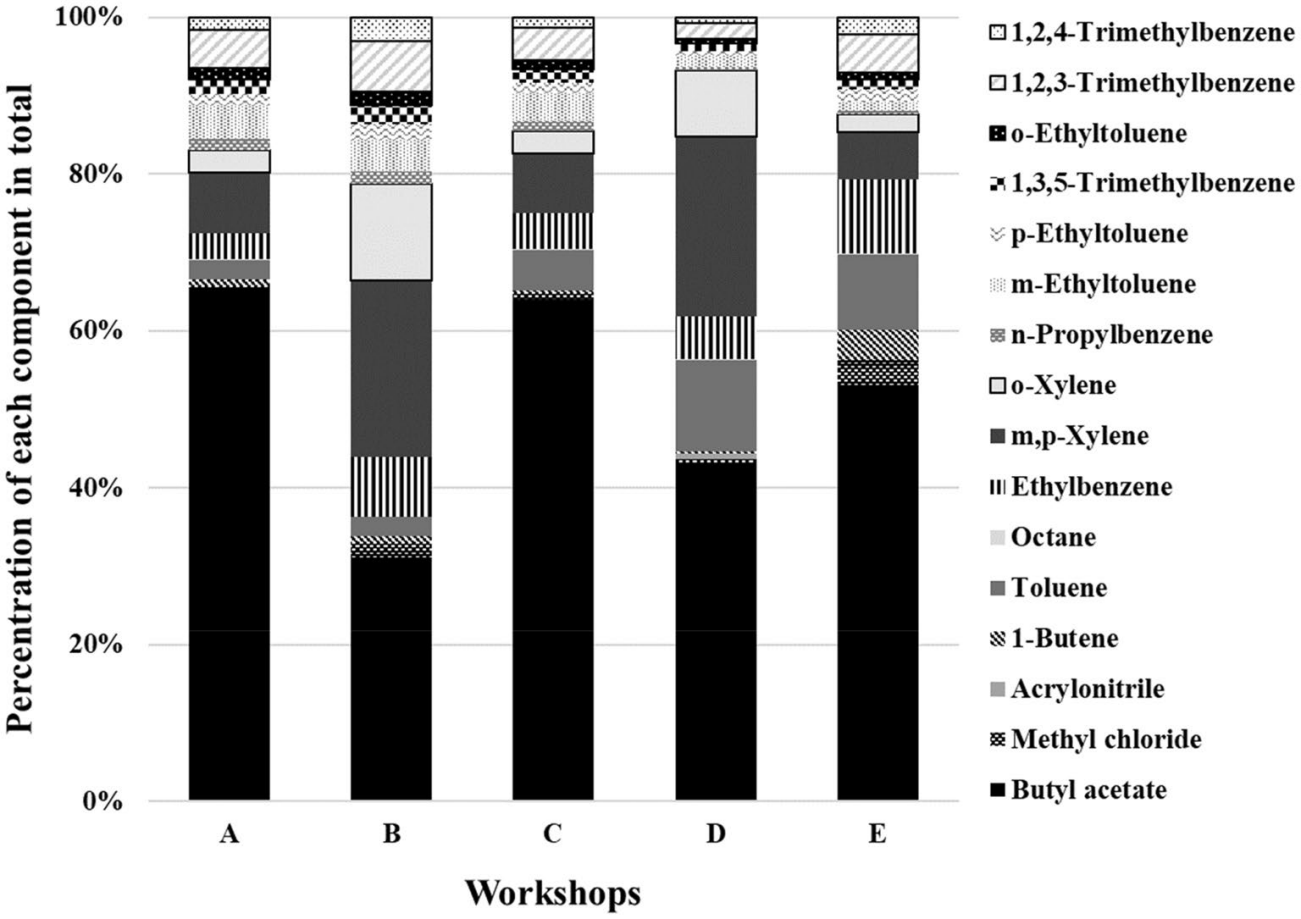
Proportion of volatile organic compound (VOC) species in different workshops during auto-repair painting.

The VOC mass concentration at the inlet of the adsorption systems varied considerably, possibly due to emissions from previous operations or leaks in the process equipment. The results are expressed as the fraction of each species relative to the TVOC concentration to account for differences in the VOC composition patterns between the workshops. The proportions of the VOCs in each workshop were calculated (Fig. 1). As shown in Fig. 1, when comparing workshops using water-based paint (A, C, and E) and oil-based paint (B and D), similar VOC composition patterns were found depending on the type of paint. In workshops A, C, and E, the concentration of butyl acetate was > 50%, while that of all the other compounds was < 10%. On the contrary, in workshops B and D, butyl acetate, m,p-xylene, o-xylene, and toluene were evenly detected at concentrations of 10% or higher. Workshops A, C, and E used water-based paint, whereas B and D used oil-based paint; hence, the components and proportions of VOCs emitted from the painting workshops were different, as noted in previous studies^25,26^. In particular, the VOCs emitted from workshop A comprised butyl acetate (65.5%), m,p-xylene (7.8%), 1,2,3-trimethylbenzene (4.9%), m-ethyltoluene (4.2%), ethylbenzene (3.2%), o-xylene (2.9%), and toluene (2.4%). In contrast, the VOCs emitted from workshop B, which used oil-based paint, comprised butyl acetate (31.1%), m,p-xylene (22.7%), o-xylene (12.5%), ethylbenzene (7.6%), 1,2,3-trimethylbenzene (6.5%), m-ethyltoluene (4.0%), and 1,2,4-trimethylbenzene (3.0%). Therefore, we next compared and analyzed the detailed characteristics of VOCs emitted from the use of water-based and oil-based paints.

### Analysis of VOCs emitted from painting workshops based on the use of different painting solvents

Information on the type of paint, type of working process, mixing ratio, amount of paint used, and working time of each workshop is summarized in Table 2. The total working time and working process were the same for workshops A and B, but the mixing ratio and amount of paint used were different. In the base coat and top coat workshops, the amount of paint used in workshop A was 847.8 g, which was higher by 325.0 g than that used in workshop B. As for the mixing ratio, the ratio of paint to diluent in the top coat used in workshop A was higher than that used in workshop B; the total working time was the same, but the base coat time in workshop A was 6 min shorter than that in workshop B.

**Table 2:** Characteristics of two different auto-repair painting workshops.

Paint type	Working process		Mixing ratio	Amount of paint used (g)	Working time (min)	
**Workshop A**						
Water-based	Base coat	Paint	4	243	16	26
		Diluent	1	60.8		
	Top coat	Paint	3	408	10	
		Diluent	1	136		
**Workshop B**						
Oil-based	Base coat	Paint	4	167	22	26
		Diluent	1	41.8		
	Top coat	Paint	2	209	4	
		Diluent	1	105		

**Figure 2:**
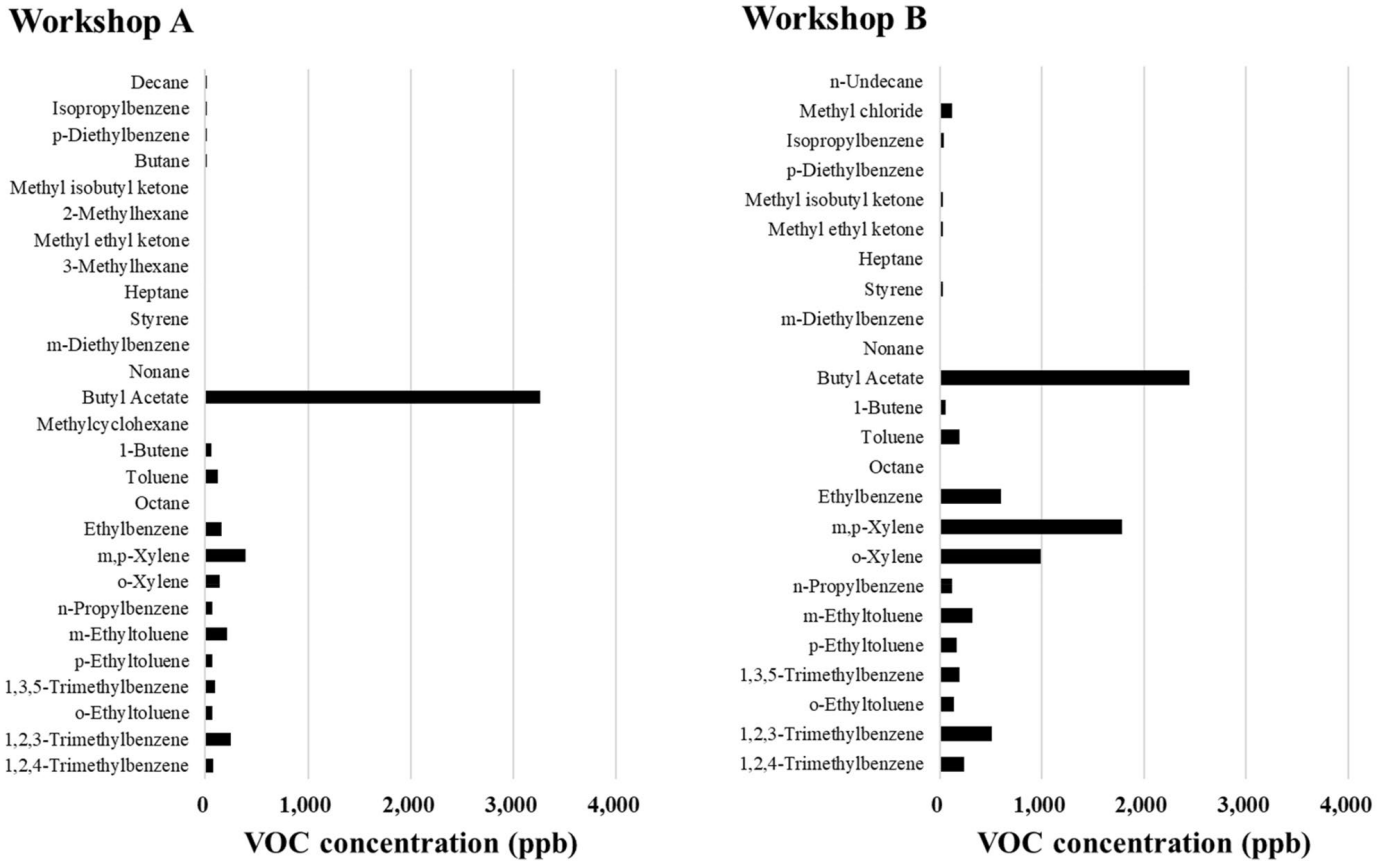
Concentration of volatile organic compounds (VOCs) emitted from workshops A and B.

The VOC emission compositions of workshops A and B from the main painting workshop are shown in Fig. 2. A similar pattern of VOC species composition was observed, but the range of concentrations was very different. The top five VOCs, in decreasing order, were butyl acetate (3256 ppb), m,p-xylene (389.1 ppb), 1,2,3-trimethybenzene (244.9 ppb), m-ethyltoluene (209.9 ppb), and ethylbenzene (106.4 ppb) in workshop A, and butyl acetate (2449 ppb), m,p-xylene (177.6 ppb), o-xylene (986.4 ppb), ethylbenzene (597.9 ppb), and 1,2,3-trimethybenzene (514.2 ppb) in workshop B. Workshop B had a higher concentration of all VOC species than workshop A, except for butyl acetate. In particular, the concentrations of m,p-xylene and o-xylene were higher in workshop B than in workshop A, which is considered to be a characteristic of oil-based painting, as xylene is the solvent in paints, thinners, and hardeners^12,27^. These results suggested that the amount of VOC emissions and concentrations of individual VOC species differed depending on the type of paint used.

### Focus on ketones and BTEX

Ketones, such as methyl ethyl ketone and methyl isobutyl ketone, are toxic chemicals that are widely used in industrial solvents^28^; inhalation of their vapors and skin contact with liquid ketones are the main routes of human exposure to these compounds^6^. Several studies have shown BTEX pollutants to have a significant adverse impact on human health and classified them as hazardous because they cause serious pathologic conditions, such as asthma, inflammatory disorders, central nervous system dysfunction, and DNA damage, which can potentially lead to cancer^29,30^. In plants, high O_3_ concentrations affect the growth and yield, whereas in human beings it has been known to exert harmful effects on the eyes and respiratory system, resulting in reduced lung capacity, respiratory distress, etc.^31,32^. Therefore, in this study, VOC species emitted from workshops A and B were grouped based on the total concentration of ketones, BTEX, and O_3_ precursors.

**Figure 3:**
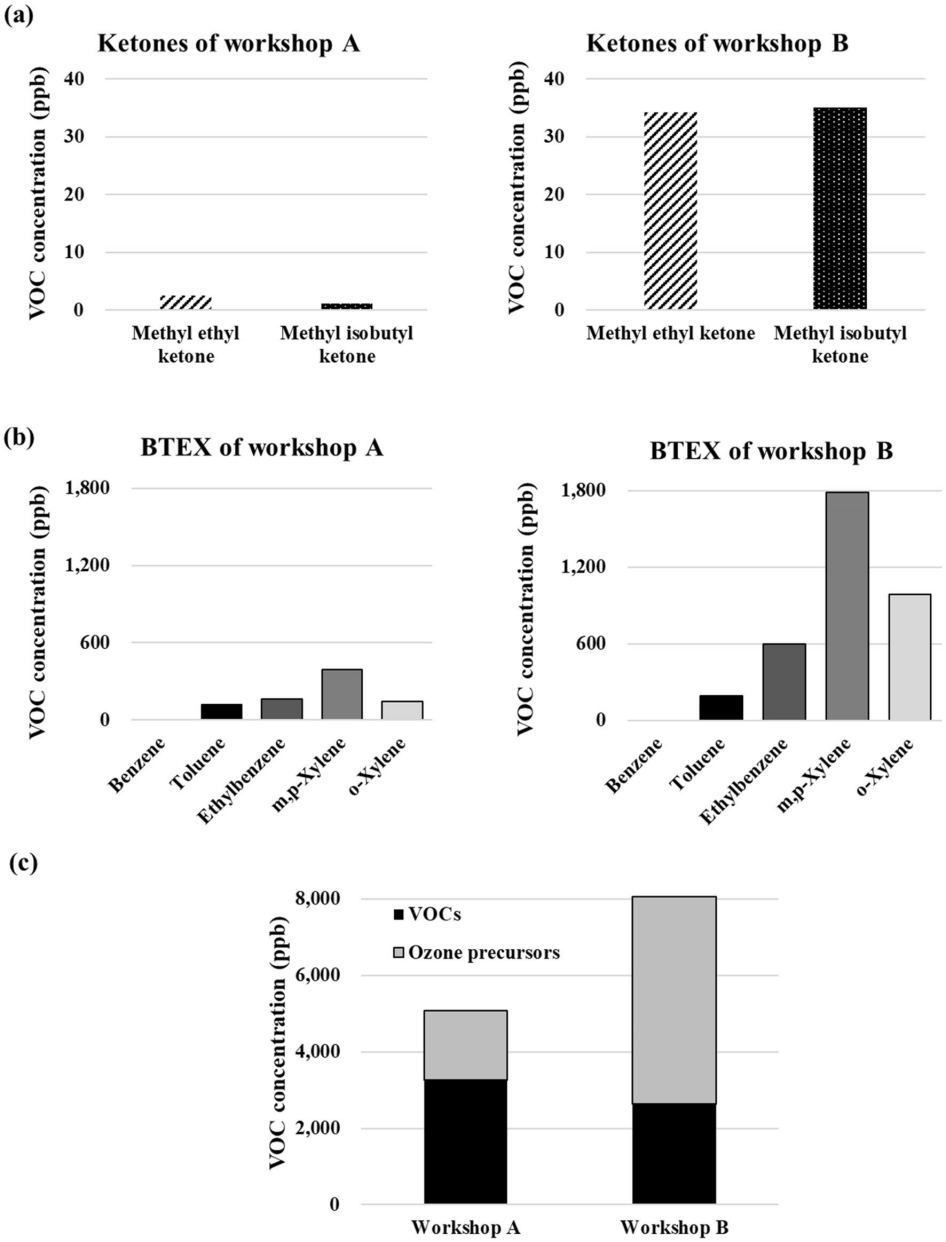
Categorization of volatile organic compounds (VOCs) emitted from workshops A and B into ketones, BTEX (benzene, toluene, ethylbenzene, and xylene), and O_3_ precursors. (**a**) Ketones emitted from workshops A and B. (**b**) BTEX emitted from workshops A and B. (**c**) Emissions from workshops A and B based on VOC classification (O_3_ precursors and VOC species).

Figure 3a shows a comparison of the ketones in workshops A and B; the concentrations of methyl ethyl ketone and methyl isobutyl ketone in workshop A were 2.554 and 1.082 ppb, respectively, while in workshop B, these were 34.29 and 35.12 ppb, respectively. Comparing the concentration of BTEX (Fig. 3b), benzene was not observed in workshops A and B, and TEX was observed at a concentration of 118.6–389.1 ppb in workshop A and 192.6–1789 ppb in workshop B. Figure 3c shows the overall TVOC emissions from workshops A and B. The VOC emissions were calculated based on the analytical group (VOCs and O_3_ precursors). Concerning the composition of VOCs emitted from the main painting workshop, workshop B (O_3_ precursors: 5421 ppb, VOCs: 2636 ppb, and TVOCs: 8058 ppb) had a higher TVOC concentration (sum of concentrations of O_3_ precursors and VOCs) than workshop A (O_3_ precursors: 1810 ppb, VOCs: 3259 ppb, and TVOCs: 5069 ppb), and the proportion of O_3_ precursors was the highest in workshop B among all VOCs. From the results shown in Figs. 1, 2, 3, we inferred that the use of oil-based paint emitted a higher concentration of harmful VOC species than that of water-based paint.

### VOC removal efficiency

Table 3 shows the measured TVOC concentrations (sum of all the VOCs) at the inlet and outlet in the booth in the top coat workshop. Workshops A, B, and E (workshop A inlet: 5069 ppb, outlet: 1492 ppb; workshop B inlet: 8058 ppb, outlet: 6187 ppb; and workshop E inlet: 6547 ppb, outlet: 6404 ppb) showed lower VOC concentrations at the outlet than at the inlet, whereas workshops C and D (workshop C inlet: 4877 ppb, outlet: 6669 ppb; and workshop D inlet: 6703 ppb; outlet: 7097 ppb) had higher VOC concentrations at the outlet than at the inlet.

**Table 3:** Total volatile organic compound (TVOC) concentrations at the inlet and outlet of the painting booths during topcoat application (unit: ppb).

	Workshop A		Workshop B		Workshop C		Workshop D		Workshop E	
TVOCs	Inlet	Outlet	Inlet	Outlet	Inlet	Outlet	Inlet	Outlet	Inlet	Outlet
	5069	1492	8058	6187	4877	6669	6703	7097	6547	6404

Supplementary Table S2 shows the concentrations of the 10 main components of VOCs emitted in the booth. The concentrations of the VOCs measured at the outlet were observed to be lower than those of the VOCs emitted from the inlet, but toluene (workshop A inlet: 118.6 ppb, outlet: 294.3 ppb; and workshop B inlet: 192.6 ppb, outlet: 200.2 ppb) was confirmed to exhibit negative efficiencies. This is considered to be due to adsorption systems not being replaced, and detachment of adsorbent or decreased absorption efficiency of activated carbon^21^. Although most VOC species in workshops A and B had a lower outlet concentration than an inlet concentration, it was insufficient to remove the pollutants emitted from the painting workshop.

## Discussion

Recently, the Korean government implemented a policy to supply eco-friendly paints to reduce and manage VOCs emitted from paints used in auto-repair painting, which involves switching from oil-based paints to water-based paints. Therefore, our findings can be used to manage VOC emissions and prepare guidelines to minimize worker exposure to these emissions. In auto-repair painting workshops, workers are exposed to the paint (dermal exposure as well as via inhalation); hence, management is necessary to not only control VOC emissions, but also minimize worker exposure. In particular, the occupational exposure limits for BTEX have been set by the Occupational Safety and Health Administration (OSHA), National Institute for Occupational Safety and Health, and American Conference of Governmental Industrial Hygienists. Based on the OSHA standards, the legal airborne permissible exposure limit-time weighted average for benzene, toluene, ethylbenzene, and xylene is 1, 200, 100, and 100 ppm, respectively, averaged over an 8 h work shift (Supplementary Table S3).

When comparing the concentrations of BTEX emitted from water-based and oil-based paints, the BTEX concentration was higher in workshop B than in workshop A, and in particular, the concentration of m,p-xylene was about 1800 ppb in workshop B. All BTEX compounds, including xylene, did not exceed the exposure limit set by the OSHA, National Institute for Occupational Safety and Health, and American Conference of Governmental Industrial Hygienists (100 ppm, time weighted average). According to the OSHA, the face, eyes, head, hands, and all other exposed parts of the bodies of employees handling such highly volatile paints should be protected using appropriate personal protection equipment for occupational safety and health. However, in the present study, the workers only wore gas masks during painting. Hence, further study should be conducted to lower VOC exposure and improve workers’ safety.

Moreover, there is a need for a management plan to improve the efficiency of the adsorption systems installed to control VOC emission into the atmosphere from auto-repair painting facilities. In the future, we intend to investigate detailed characteristics (type of activated carbon, number of prevention facilities, thickness of activated carbon, replacement cycle, maintenance, etc.) of the adsorption systems installed in painting facilities to establish efficient VOC removal in the field.

## Conclusion

The concentration trends of VOC species emitted from auto-repair painting workshops in Korea were identified. In addition, we compared the detailed characteristics of the VOC species emitted from water- and oil-based paints and evaluated the VOC removal efficiency of the adsorption systems. The main components of the VOC mixture were butyl acetate, methyl chloride, acrylonitrile, 1-butene, toluene, octane, ethylbenzene, m,p-xylene, o-xylene, n-propylbenzene, m-ethyltoluene, p-ethyltoluene, 1,3,5-trimethylbenzene, o-ethyltoluene, 1,2,3-trimethylbenzene and 1,2,4-trimethylbenzene; butyl acetate, toluene, m,p-xylene, o-xylene and ethylbenzene were present at relatively high concentrations. The total number of VOC species detected was 24–35. The concentrations of most VOCs emitted from oil-based paints were higher than those of VOCs emitted from water-based paints. In particular, the oil-based paints showed high concentrations of m,p-xylene and o-xylene; individual VOC species were also detected in higher proportions and concentrations from the use of oil-based paints than water-based paints. The concentrations of VOCs emitted from the outlet of the adsorption system were lower than those of VOCs emitted from the inlet; however, some workshops showed higher VOC concentrations at the outlet than the inlet.

Our findings provide a theoretical basis for managing and developing effective VOC control systems to remove the major VOCs emitted from auto-repair painting, and establishing standards for VOC content in paint application-based products. Our study could lead to further establishment of profiles of the main VOC species, which can be used to establish efficient VOC control strategies in the future.

## Methods

### Experimental set up

**Figure 4:**
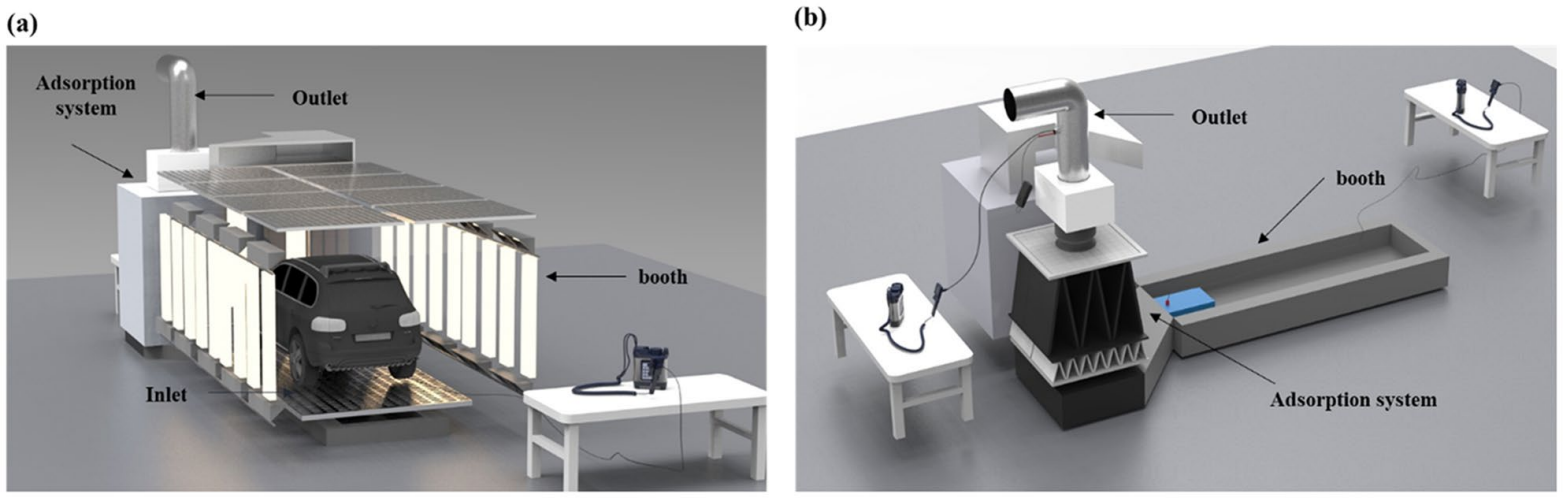
Illustration of an auto-repair painting workshop. (**a**) Illustration of a booth in an auto-repair painting workshop (Front view). (**b**) Illustration of an adsorption system connected to a booth (Top view).

**Figure 5:**
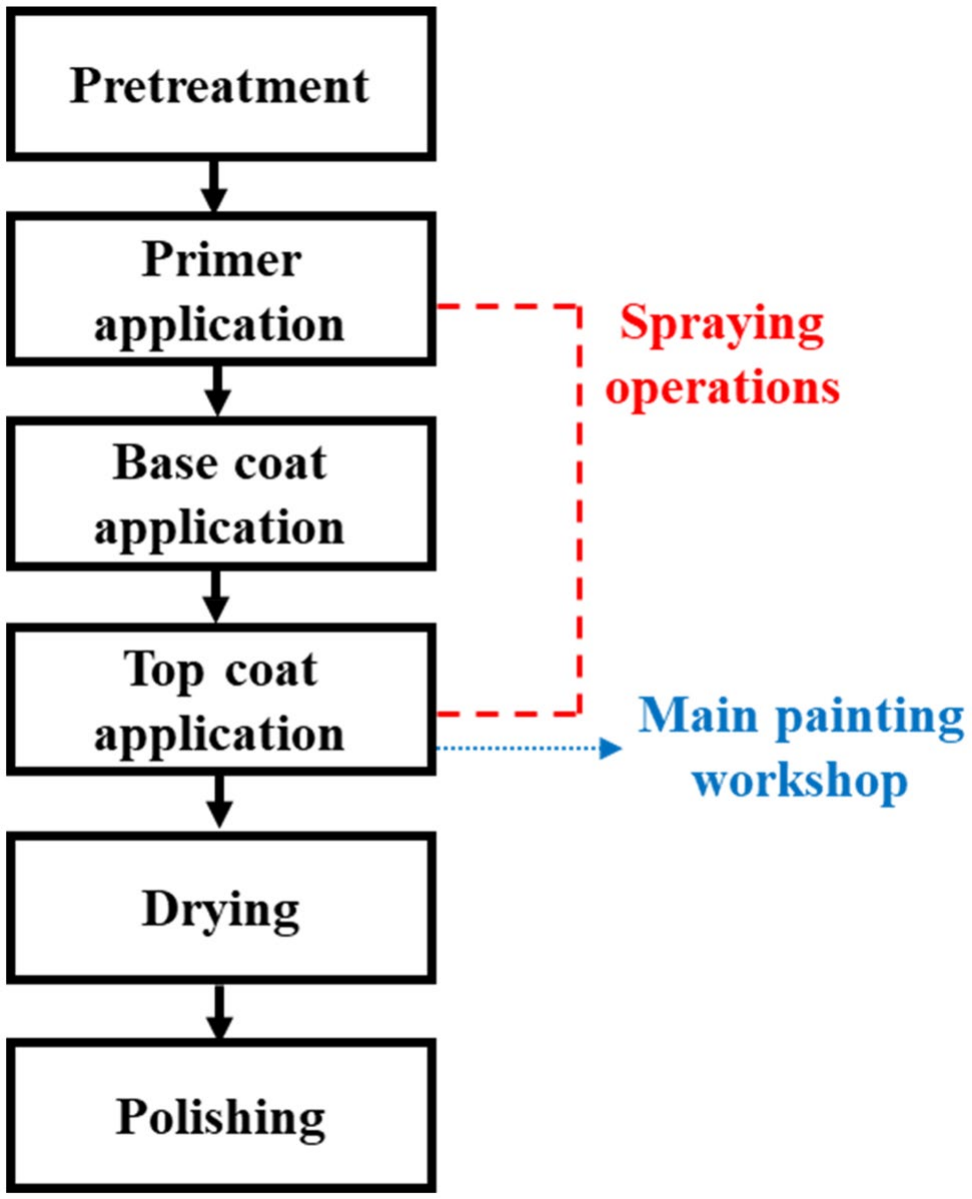
Flow chart outlining the auto-repair painting processes.

Auto-repair painting facilities (workshops A–E) in Seoul were considered in our study. Workshops B and D used oil-based paint, while workshops A, C, and E used water-based paint for auto-repair painting. All auto-repair painting facilities were equipped with an adsorption system for VOC control connected to painting booths (Fig. 4a)^33^. The painting operations occurred within the painting booth, and air samples were collected at the inlet and outlet during the main painting workshop to analyze the concentration of individual VOCs (Fig. 4b)^33^. The measurements were conducted throughout the auto-repair painting operations. In general, auto-repair painting operations start with the primer application, followed by base coat and top coat applications, and finally, drying of the automobile surface (Fig. 5).

### Measurements and analytical method

For the analysis of VOC components, sampling was conducted as per the solid adsorption method using Tenax-TA (40/60 mesh, Markers, USA) 280 mg sorbent tubes of stainless steel. The tubes were heated for 6 h at 300 °C using a TC-20 device (Markes, USA) prior to the measurements. VOC sampling was conducted for 5 min during the painting and drying processes; the air collection rate was 100 mL/min. The flow rate was corrected using a sampling pump equipped with a sorbent tube, before starting the measurement. Tenax-TA sorbent tubes were refrigerated at 4 °C or below and analyzed using GC/MSD (Agilent HP-6890, USA). As VOC standards, 10 CHEM and 50 ozone precursors were analyzed, and SUPELCO's TO-14 VOC standard mixture (nominal 1 ppm) containing 42 toxic VOCs was used (Supplementary Table S4). Out of a total of 102 chemicals, 88 substances were classified by excluding overlapping substances.

The gas chromatography (GC)/mass spectrometry (MS) analysis conditions (Supplementary Table S5) were as follows: secondary thermal desorption from the thermal desorption device; solvent delay performed for 5 min to minimize analysis of the initial low molecular material; initial temperature of the GC oven maintained at 50 °C for 10 min, gradually increased to 220 °C, and maintained for 10 min; and the post run set to 5 min to reduce the contamination of the equipment due to the inflow of polymers other than the material to be measured. The analysis time was approximately 54 min.

To evaluate the linearity of VOC concentrations, a liquid standard (100 μg/mL; Supelco, USA) was added in small amounts at 100, 300, and 500 ng to three Tenax-TA-using adsorbent tube injector systems (Supelco, USA), respectively, and then, after thermal desorption using Autosampler Thermal Desorption (Ultra-xr, Markes), a calibration curve for VOCs was plotted.

Evaluation of the linearity of the calibration curve revealed the coefficient of determination (R^2^) as 0.99 or higher and the relative standard deviation, which represents reproducibility of the analysis, was evaluated to range from 0.52 to 4.32% as a result of seven repeated analyses.

## Supplementary Information

Supplementary Information.
