# Supplementary material for: Species and characteristics of volatile organic compounds emitted from an auto-repair painting workshop
Source: Sci Rep. 2021 Aug 16;11:16586. doi: 10.1038/s41598-021-96163-4 (PMC8368184; doi:10.1038/s41598-021-96163-4)
Supplement: Supplementary file 1 — Supplementary Information. [file 41598_2021_96163_MOESM1_ESM.docx]

**Species and Characteristics of Volatile Organic Compounds Emitted from an Auto-Repair Painting Workshop**

M Y Song^a*^, H Chun ^a^

**Supplementary Information**

**Supplementary Figure S1.** Volatile organic compound (VOC) standard chromatograms obtained via gas chromatography-mass spectrometry analysis.

| 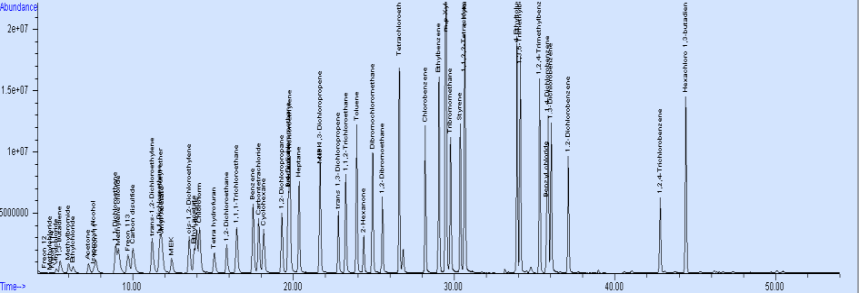 |
| --- |
| 1. TO-14 standard chromatogram. |
| 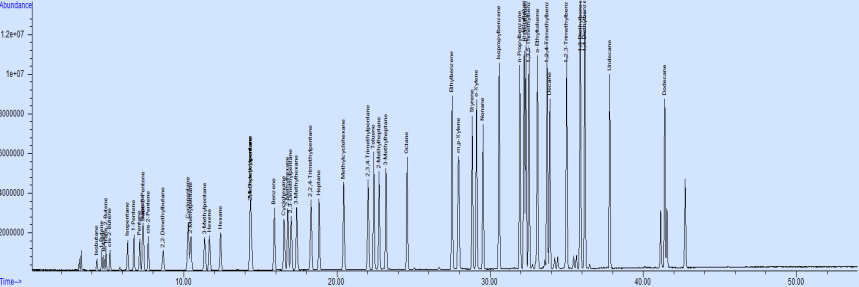 |
| 1. Ozone precursor standard chromatogram. |
|  |

**Supplementary Table S1.** Analytical targets and concentrations of volatile organic compounds (VOCs).

| Limits of  quantification | Compounds | Classification | Workshop A | Workshop B | Workshop C | Workshop D | Workshop E |
| --- | --- | --- | --- | --- | --- | --- | --- |
| 0.09 | Methyl ethyl ketone | CHEM | 2.554 | 34.29 | 1.928 | 38.74 | 6.205 |
| 0.13 | Isobutyl alcohol |  | 0.000 | 0.000 | 0.000 | 11.19 | 78.41 |
| 0.06 | Methyl isobutyl ketone |  | 1.082 | 35.12 | 2.078 | 218.6 | 28.04 |
| 0.05 | Butyl Acetate |  | 3,256 | 2,449 | 3,054 | 2,581 | 3,378 |
| 0.01 | Isopropyl alcohol |  | 0.000 | 0.000 | 0.000 | 119.87 | 0.000 |
| 0.05 | Freon 12 | TO-14 | 0.000 | 0.000 | 0.000 | 0.000 | 0.000 |
| 0.04 | Methyl chloride |  | 0.000 | 117.6 | 38.01 | 30.61 | 211.9 |
| 0.06 | Freon 114 |  | 0.000 | 0.000 | 0.000 | 0.000 | 0.000 |
| 0.04 | Vinyl chloride |  | 0.000 | 0.000 | 0.000 | 0.000 | 0.000 |
| 0.09 | 1,3-Butadiene |  | 0.000 | 0.000 | 0.000 | 0.000 | 0.000 |
| 0.10 | Methyl bromide |  | 0.000 | 0.000 | 0.000 | 0.000 | 0.000 |
| 0.10 | Ethylchloride |  | 0.000 | 0.000 | 0.000 | 0.000 | 0.000 |
| 0.10 | Freon 11 |  | 0.000 | 0.000 | 0.000 | 0.000 | 0.000 |
| 0.08 | Acrylonitrile |  | 0.000 | 0.000 | 0.000 | 0.000 | 0.000 |
| 0.07 | 1,1-Dichloroethene |  | 0.000 | 0.000 | 0.000 | 0.000 | 0.000 |
| 0.10 | Dichloromethane |  | 0.000 | 0.000 | 0.000 | 12.07 | 15.62 |
| 0.11 | 3-Chloropropylene |  | 0.000 | 0.000 | 0.000 | 0.000 | 0.000 |
| 0.11 | Freon 113 |  | 0.000 | 0.000 | 0.000 | 0.000 | 0.000 |
| 0.05 | 1,1-Dichloroethane |  | 0.000 | 0.000 | 0.000 | 0.000 | 0.000 |
| 0.05 | cis-1,2-Dichloroethylene |  | 0.000 | 0.000 | 0.000 | 0.000 | 0.000 |
| 0.03 | Chloroform |  | 0.000 | 0.000 | 0.000 | 0.000 | 9.883 |
| 0.10 | 1,2-Dichloroethane |  | 0.000 | 0.000 | 0.000 | 0.000 | 0.000 |
| 0.12 | Methyl chloroform |  | 0.000 | 0.000 | 0.000 | 0.000 | 0.000 |
| 0.09 | Carbontetrachloride |  | 0.000 | 0.000 | 0.000 | 0.000 | 0.000 |
| 0.12 | 1,2-Dichloropropane |  | 0.000 | 0.000 | 0.000 | 0.000 | 0.000 |
| 0.09 | Trichloroethylene |  | 0.000 | 0.000 | 0.000 | 0.000 | 0.000 |
| 0.08 | cis 1,3-Dichloropropene |  | 0.000 | 0.000 | 0.000 | 0.000 | 0.000 |
| 0.13 | trans 1,3-Dichloropropene |  | 0.000 | 0.000 | 0.000 | 0.000 | 0.000 |
| 0.14 | 1,1,2-Trichloroethane |  | 0.000 | 0.000 | 0.000 | 0.000 | 0.000 |
| 0.13 | 1,2-Dibromoethane |  | 0.000 | 0.000 | 0.000 | 0.000 | 0.000 |
| 0.04 | Tetrachloroethylene |  | 0.000 | 0.000 | 0.000 | 0.000 | 0.000 |
| 0.14 | Chlorobenzene |  | 0.000 | 0.000 | 0.000 | 0.000 | 0.000 |
| 0.19 | 1,1,2,2-Tetrachloroethane |  | 0.000 | 0.000 | 0.000 | 0.000 | 0.000 |
| 0.15 | m-Dichlorobenzene |  | 0.000 | 0.000 | 0.000 | 0.000 | 0.000 |
| 0.11 | p-Dichlorobenzene |  | 0.000 | 0.000 | 0.000 | 0.000 | 0.000 |
| 0.10 | o-Dichlorobenzene |  | 0.000 | 0.000 | 0.000 | 0.000 | 0.000 |
| 0.16 | 1,2,4-Trichlorobenzene |  | 0.000 | 0.000 | 0.000 | 0.000 | 0.000 |
| 0.19 | Hexachloro 1,3-butadiene |  | 0.000 | 0.000 | 0.000 | 0.000 | 0.000 |
| 0.10 | Isobutane | Ozone precursors | 0.000 | 0.000 | 0.000 | 0.000 | 0.000 |
| 0.09 | 1-Butene |  | 55.66 | 60.42 | 14.30 | 16.10 | 244.2 |
| 0.07 | Butane |  | 17.33 | 0.000 | 0.000 | 9.159 | 0.000 |
| 0.08 | trans-2-Butene |  | 0.000 | 0.000 | 0.000 | 0.000 | 0.000 |
| 0.09 | cis-2-Butene |  | 0.000 | 0.000 | 0.000 | 0.000 | 0.000 |
| 0.04 | Isopentane |  | 0.000 | 0.000 | 0.000 | 0.000 | 0.000 |
| 0.12 | 1-Pentene |  | 0.000 | 0.000 | 0.000 | 0.000 | 0.000 |
| 0.04 | Pentane |  | 0.000 | 0.000 | 0.000 | 0.000 | 0.000 |
| 0.11 | Isoprene |  | 0.000 | 0.000 | 0.000 | 0.000 | 0.000 |
| 0.08 | trans-2-Pentene |  | 0.000 | 0.000 | 0.000 | 0.000 | 0.000 |
| 0.06 | cis-2-Pentene |  | 0.000 | 0.000 | 0.000 | 0.000 | 0.000 |
| 0.05 | 2,2-Dimethylbutane |  | 0.000 | 0.000 | 0.000 | 0.000 | 0.000 |
| 0.10 | Cyclopentane |  | 0.000 | 0.000 | 0.000 | 0.000 | 0.000 |
| 0.13 | 2-Methylpentane |  | 0.000 | 0.000 | 0.000 | 0.000 | 0.000 |
| 0.13 | 3-Methylpentane |  | 0.000 | 0.000 | 0.000 | 0.000 | 0.000 |
| 0.12 | Hexene |  | 0.000 | 0.000 | 0.000 | 0.000 | 0.000 |
| 0.09 | Hexane |  | 0.000 | 0.000 | 0.000 | 0.000 | 0.000 |
| 0.11 | Methylcyclopentane |  | 0.000 | 0.000 | 0.000 | 0.000 | 0.000 |
| 0.07 | 2,4-Dimethylpentane |  | 0.000 | 0.000 | 0.000 | 0.000 | 0.000 |
| 0.07 | Benzene |  | 0.000 | 0.000 | 0.000 | 0.525 | 2.316 |
| 0.11 | Cyclohexane |  | 0.965 | 0.000 | 1.352 | 0.000 | 1.284 |
| 0.14 | 2-Methylhexane |  | 2.086 | 0.000 | 2.731 | 1.316 | 2.200 |
| 0.08 | 2,3-Dimethylpentane |  | 0.768 | 0.000 | 1.093 | 0.000 | 0.000 |
| 0.03 | 3-Methylhexane |  | 2.763 | 0.000 | 4.324 | 1.800 | 2.420 |
| 0.06 | 2,2,4-Trimethylpentane |  | 0.000 | 0.000 | 0.000 | 0.000 | 0.000 |
| 0.05 | Heptane |  | 3.254 | 8.255 | 5.086 | 3.276 | 10.59 |
| 0.05 | Methylcyclohexane |  | 2.593 | 0.000 | 2.906 | 1.411 | 1.761 |
| 0.03 | 2,3,4-Trimethylpentane |  | 0.000 | 0.000 | 0.000 | 0.000 | 0.000 |
| 0.08 | Toluene |  | 118.6 | 192.6 | 246.5 | 689.8 | 613.8 |
| 0.06 | 2-Methylheptane |  | 0.000 | 0.000 | 3.585 | 2.525 | 0.000 |
| 0.07 | 3-Methylheptane |  | 0.000 | 0.000 | 2.792 | 0.000 | 0.000 |
| 0.03 | Octane |  | 8.756 | 4.759 | 8.465 | 8.381 | 14.08 |
| 0.04 | Ethylbenzene |  | 160.4 | 597.9 | 215.1 | 325.4 | 599.1 |
| 0.03 | m,p-Xylene |  | 389.1 | 1,789 | 367.2 | 1,370 | 383.1 |
| 0.04 | Styrene |  | 5.273 | 27.77 | 12.47 | 11.98 | 94.03 |
| 0.07 | o-Xylene |  | 143.6 | 986.4 | 132.5 | 507.7 | 151.5 |
| 0.03 | Nonane |  | 8.649 | 7.514 | 10.79 | 48.33 | 7.155 |
| 0.02 | Isopropylbenzene |  | 14.24 | 38.02 | 13.55 | 17.87 | 29.63 |
| 0.01 | n-Propylbenzene |  | 70.85 | 123.2 | 61.98 | 31.13 | 27.39 |
| 0.02 | m-Ethyltoluene |  | 209.9 | 316.3 | 173.0 | 79.31 | 66.12 |
| 0.02 | p-Ethyltoluene |  | 69.28 | 164.0 | 61.77 | 40.72 | 100.4 |
| 0.03 | 1,3,5-Trimethylbenzene |  | 94.45 | 190.4 | 78.22 | 56.81 | 83.04 |
| 0.01 | o-Ethyltoluene |  | 70.92 | 138.4 | 58.88 | 32.58 | 56.46 |
| 0.06 | 1,2,3-Trimethylbenzene |  | 244.9 | 514.2 | 194.1 | 122.5 | 316.5 |
| 0.03 | Decane |  | 12.11 | 0.000 | 12.41 | 41.83 | 13.45 |
| 0.05 | 1,2,4-Trimethylbenzene |  | 80.77 | 239.3 | 66.98 | 45.59 | 137.6 |
| 0.03 | m-Diethylbenzene |  | 8.166 | 5.883 | 6.302 | 4.115 | 7.135 |
| 0.08 | p-Diethylbenzene |  | 14.86 | 12.40 | 17.85 | 9.137 | 9.551 |
| 0.08 | n-Undecane |  | 0.000 | 4.663 | 4.793 | 11.75 | 0.000 |
| 0.08 | n-Dodecane |  | 0.000 | 0.000 | 0.000 | 0.000 | 0.000 |

**Supplementary Table S2.** Concentrations of various volatile organic compound (VOC) species at the inlet and outlet of the booths for water-based (workshop A) and oil-based (workshop B) painting (unit: ppb).

| Compounds | Workshop A | | Workshop B | |
| --- | --- | --- | --- | --- |
|  | Inlet | Outlet | Inlet | Outlet |
| Butyl Acetate | 3,256 | 622.0 | 2,449 | 2,169 |
| Toluene | 118.6 | 294.3 | 192.6 | 200.2 |
| Ethylbenzene | 160.4 | 134.4 | 597.9 | 88.93 |
| m,p-Xylene | 389.1 | 85.52 | 1,789 | 1,531 |
| o-Xylene | 143.6 | 32.25 | 986.4 | 645.0 |
| m-Ethyltoluene | 209.9 | 10.45 | 316.3 | 186.6 |
| p-Ethyltoluene | 69.28 | 10.43 | 164.0 | 98.11 |
| 1,3,5-Trimethylbenzene | 94.45 | 9.560 | 190.4 | 112.5 |
| 1,2,3-Trimethylbenzene | 244.9 | 37.94 | 514.2 | 310.4 |
| 1,2,4-Trimethylbenzene | 80.77 | 27.68 | 239.3 | 150.1 |

**Supplementary Table S3.** Volatile organic compound exposure limits set by different organizations.

|  | OSHA-PEL^1)^ | | | NIOSH-REL^2)^ | | | ACGIH-TLV^3)^ | | | |
| --- | --- | --- | --- | --- | --- | --- | --- | --- | --- | --- |
| Compound | TWA^4)^ (ppm) | STEL^5)^ (ppm) | C^6)^  (ppm) | TWA (ppm) | STEL (ppm) | C (ppm) | TWA (ppm) | STEL (ppm) | C (ppm) |  |
| Benzene | 1 | 5 |  | 0.1 | 1 |  | 0.5 | 2.5 |  |  |
| Toluene | 200 |  | 300 | 100 | 150 |  | 20 |  |  |  |
| Ethylbenzene | 100 |  |  | 100 | 125 |  | 20 |  |  |  |
| Xylene | 100 |  |  | 100 | 150 |  | 100 | 150 |  |  |
| ^1)^Occupational Safety and Health Administration (OSHA) - Permissible Exposure Limit (PEL) | | | | | | | | | | |
| ^2)^National Institute for Occupational Safety and Health (NIOSH) - Recommended Exposure Limit (REL) | | | | | | | | | | |
| ^3)^American Conference of Governmental and Industrial Hygienists (ACGIH) - Threshold Limit Value (TLV) | | | | | | | | | | |
| ^4)^Time Weighted Average (TWA)  ^5)^Short-term Exposure Limit (STEL)  ^6)^Ceiling | | | | | | | | | | |

**Supplementary Table S4.** Detailed list of volatile organic compounds analyzed in the study.

| TO-14 | | Ozone precursors | | CHEM | |
| --- | --- | --- | --- | --- | --- |
| No. | Chemicals | No. | Chemicals | No. | Chemicals |
| 1 | Freon 12 | 1 | Isobutane | 1 | Methyl ethyl ketone |
| 2 | Methylchloride | 2 | 1-Butene | 2 | Isobutyl alcohol |
| 3 | Freon 114 | 3 | Butane | 3 | Benzene |
| 4 | Vinylchloride | 4 | trans-2-Butene | 4 | Methyl isobutyl ketone |
| 5 | 1,3-Butadiene | 5 | cis-2-Butene | 5 | Toluene |
| 6 | Methylbromide | 6 | Isopentane | 6 | Butyl Acetate |
| 7 | Ethylchloride | 7 | 1-Pentene | 7 | Ethylbenzene |
| 8 | Freon 11 | 8 | Pentane | 8 | m,p-Xylene |
| 9 | Acrylonitrile | 9 | Isoprene | 9 | Styrene |
| 10 | 1,1-Dichloroethene | 10 | trans-2-Pentene | 10 | o-Xylene |
| 11 | Dichloromethane | 11 | cis-2-Pentene |  |  |
| 12 | 3-Chloropropylene | 12 | 2,2-Dimethylbutane |  |  |
| 13 | Freon 113 | 13 | Cyclopentane |  |  |
| 14 | 1,1-Dichloroethane | 14 | 2-Methylpentane |  |  |
| 15 | cis-1,2-Dichloroethylene | 15 | 3-Methylpentane |  |  |
| 16 | Chloroform | 16 | 1-Hexene |  |  |
| 17 | 1,2-Dichloroethane | 17 | 2-Hexane |  |  |
| 18 | Methyl chloroform | 18 | Methylcyclopentane |  |  |
| 19 | Benzene | 19 | 2,4-Dimethylpentane |  |  |
| 20 | Carbontetrachloride | 20 | Benzene |  |  |
| 21 | 1,2-Dichloropropane | 21 | Cyclohexane |  |  |
| 22 | Trichloroethylene | 22 | 2-Methylhexane |  |  |
| 23 | cis 1,3-Dichloropropene | 23 | 2,3-Dimethylpentane |  |  |
| 24 | trans 1,3-Dichloropropene | 24 | 3-Methylhexane |  |  |
| 25 | 1,1,2-Trichloroethane | 25 | 2,2,4-Trimethylpentane |  |  |
| 26 | Toluene | 26 | Heptane |  |  |
| 27 | 1,2-Dibromoethane | 27 | Methylcyclohexane |  |  |
| 28 | Tetrachloroethylene | 28 | 2,3,4-Trimethylpentane |  |  |
| 29 | Chlorobenzene | 29 | Toluene |  |  |
| 30 | Ethylbenzene | 30 | 2-Methylheptane |  |  |
| 31 | m,p-Xylene | 31 | 3-Methylheptane |  |  |
| 32 | Styrene | 32 | Octane |  |  |
| 33 | 1,1,2,2-Tetrachloroethane | 33 | Ethylbenzene |  |  |
| 34 | o-Xylene | 34 | m,p-Xylene |  |  |
| 35 | 1-Ethyl-4-methylbenzene | 35 | Styrene |  |  |
| 36 | 1,3,5-Trimethylbenzene | 36 | o-Xylene |  |  |
| 37 | 1,2,4-Trimethylbenzene | 37 | Nonane |  |  |
| 38 | m-Dichlorobenzene | 38 | Isopropylbenzene |  |  |
| 39 | p-Dichlorobenzene | 39 | n-Propylbenzene |  |  |
| 40 | o-Dichlorobenzene | 40 | m-Ethyltoluene |  |  |
| 41 | 1,2,4-Trichlorobenzene | 41 | p-Ethyltoluene |  |  |
| 42 | Hexachloro 1,3-butadiene | 42 | 1,3,5-Trimethylbenzene |  |  |
| 43 |  | 43 | o-Ethyltoluene |  |  |
| 44 |  | 44 | 1,2,4-Trimethylbenzene |  |  |
| 45 |  | 45 | Decane |  |  |
| 46 |  | 46 | 1,2,3-Trimethylbenzene |  |  |
| 47 |  | 47 | m-Diethylbenzene |  |  |
| 48 |  | 48 | p-Diethylbenzene |  |  |
| 49 |  | 49 | n-Undecane |  |  |
| 50 |  | 50 | n-Dodecane |  |  |

Highlight: overlapping compounds

**Supplementary Table S5.** Operation conditions for thermal desorption and gas chromatography-mass spectrometry (GC/MS) analysis.

| Thermal desorption | | GC/MS system | |
| --- | --- | --- | --- |
| Apparatus | Operation conditions | Apparatus | Operation conditions |
| Flow path temp. | 120 °C | Column | DB-1(60 m × 0.32 mm × 3 μm) |
| Prepurge time | 1.0 min | Initial temp. | 50 °C (10 min) |
| Desorb temp. | 300 °C | Final temp. | 220 °C (10 min) |
| Desorb time | 10 min | Ramp rate | 5 °C/min |
| Trap flow | 50 mL/min | Column flow | 1.2 mL/min |
| Trap purge time | 1 min | Detector type | Quadropole |
| Trap purge flow | 30 mL/min | Q-pole temp. | 150 °C |
| Trap low temp. | -20 °C | MS source temp. | 230 °C |
| Trap high temp. | 300 °C |  |  |
| Trap desorb time | 4 min |  |  |
| Split flow | 15 mL/min |  |  |
| Cold trap | Carbopack b  Carboxen 1,000 (1:1) |  |  |

Temp., temperature
